# Letter Position Dyslexia in Arabic: From Form to Position

**DOI:** 10.3233/BEN-2012-119004

**Published:** 2012-03-19

**Authors:** Naama Friedmann, Manar Haddad-Hanna

**Affiliations:** Language and Brain LabSchool of EducationTel Aviv UniversityTel AvivIsrael

**Keywords:** Dyslexia, letter position dyslexia, Arabic, abstract letter identity, developmental dyslexia

## Abstract

This study reports the reading of 11 Arabic-speaking individuals with letter position dyslexia (LPD), and the effect of letter form on their reading errors. LPD is a peripheral dyslexia caused by a selective deficit to letter position encoding in the orthographic-visual analyzer, which results in migration of letters within words, primarily of middle letters. The Arabic orthography is especially interesting for the study of LPD because Arabic letters have different forms in different positions in the word. As a result, some letter position errors require letter form change. We compared the rate of letter migrations that change letter form with migrations that do not change letter form in 10 Arabic-speaking individuals with developmental LPD, and one bilingual Arabic and Hebrew-speaking individual with acquired LPD. The results indicated that the participants made 40% letter position errors in migratable words when the resulting word included the letters in the same form, whereas migrations that changed letter form almost never occurred. The error rate of the Arabic-Hebrew bilingual reader was smaller in Arabic than in Hebrew. However, when only words in which migrations do not change letter form were counted, the rate was similar in Arabic and Hebrew. Hence, whereas orthographies with multiple letter forms for each letter might seem more difficult in some respects, these orthographies are in fact easier to read in some forms of dyslexia. Thus, the diagnosis of LPD in Arabic should consider the effect of letter forms on migration errors, and use only migratable words that do not require letter-form change. The theoretical implications for the reading model are that letter form (of the position-dependent type found in Arabic) is part of the information encoded in the abstract letter identity, and thus affects further word recognition processes, and that there might be a pre-lexical graphemic buffer in which the checking of orthographic well-formedness takes place.

## 1. Introduction

The same exact dyslexia can manifest itself differently in different orthographies. For example, surface dyslexia is less pronounced in languages with a shallow orthography than in languages with a deep orthography; neglect dyslexia, which often affects the left side of words, causes letter omission, substitution, and addition on the beginning of words in English and Italian, but on the end of words in Hebrew, which is written from right to left. Letter position dyslexia (LPD) is a deficit in the orthographic-visual analysis system that selectively impairs the ability to encode the relative position of letters within words and causes letter position errors such as reading "form" instead of "from". LPD is more easily detectible and causes more errors in languages like Hebrew, in which middle letter transpositions frequently create other existing words. This high probability of lexical transposition in Hebrew relates to the Semitic morphology, which creates many word pairs that differ in the relative order of its three root letters, or in the position of a middle morphological letter. The lexicality of transpositions also relates to the underspecification of vowels, which creates many degrees of freedom in reading the letter sequence that results from the transposition, hence increasing the probability of migration creating an existing word.

Arabic, like Hebrew, has Semitic morphology, and, like Hebrew, its orthography does not represent (short) vowels. It is thus expected that LPD will be easily detectible in Arabic as well. However, there is another aspect of Arabic orthography that might be relevant for letter position errors: Arabic letters change their form according to their position in the word (beginning, mid-

ISSN 0953-4180/12/$27.50 © 2012 – IOS Press and the authors. All rights reserved dle, or end), and according to whether or not they ligate to letters that precede them, as some Arabic letters do not ligate to the following letter and some do. For example, the letter H is written هـ when in initial position (and in middle position, when not ligated to the previous letter), ـهـ when in middle position and ligating to the previous letter, ـه when in final position and ligating to the previous letter, and ه when final and non-ligating. This property of Arabic orthography might have an immediate effect on letter position errors, as certain positions would not be appropriate for the letter forms presented, and hence many letter position errors might be blocked, leading to a decrease in the rate of such errors.

Such considerations might be indicative of the intricate interactions between orthographies and various reading deficits. They can further inform theoretical questions regarding the reading process: which features are included in the abstract letter identity, and whether there are pre-lexical processes that check the well-formedness of input strings. Arabic provides a very good testing ground for such questions, whereas such study can obviously not be done in English and similar orthographies in which middle letters do not have position-dependent letter forms.

This study presents letter position dyslexia in Arabic for the first time, and explores whether the existence of letter forms that are determined by letter position affects letter position errors in Arabic-speaking individuals with LPD.

### *1.1. Letter form in Arabic orthography*

Arabic is written from right to left. It includes 28 letters that are written in a cursive style. All Arabic letters can be used as consonants, and three of them can also be used as long vowels (a, i, u). The short vowels are usually not represented in the orthography, except for in texts for beginning readers, in which vowels are marked by vocalization diacritics [24].

The form of each letter is determined by two factors: its position in the word – initial, middle, or final, and whether or not it ligates to the letter that precedes it. Whether or not a letter ligates to the preceding letter depends on the preceding letter: six of the Arabic letters do not ligate to the following letter: ا, د, ذ, ر, ز, و (A,D,ð,R,Z,U). The combination of position and ligation creates four letter forms: a form for letters in the beginning of the word, a form for letters in the middle of the word that ligate to the preceding letter, a form for final letters that ligate to the preceding letter, and a form for final letters that do not ligate to the preceding letter. Middle letters that do not ligate to the preceding letter are written using the initial letter form.

As shown in Table 1, twenty letters change their form between initial/medial and final positions, and 8 letters only change their ligation according to whether or not they are ligated to the preceding letter.

This property of Arabic orthography whereby letter form is a product of letter position and ligation yields the following with respect to letter position errors: for some target words, a letter position error creates a word with the exact same letter forms, only in different positions (like تهمل – تمهل, TMHL-THML in orthographic transliteration, in which the M and the H exchange positions but keep their form). For other target words, however, letter position errors create a word with different letter forms, as is the case when a ligated letter moves to a position after a non-ligating letter (like جهاز - جاهز, GAHZ-GHAZ, in which the H alternates between middle-ligating and middle non ligating [initial] forms). In these cases, the same letter has different forms in different positions, and hence, transposing the letters in the middle of the word while keeping their original letter forms would create an orthographically illegal sequence, جاـهز (with an H in a ligating form after a non-ligating letter, which should have been جاهز). Such a sequence is impossible in all common Arabic fonts.

The main research question of this study was, then, whether the existence of such position-dependent letter forms affects the rate of letter position errors in LPD. Specifically, we wanted to know whether there would be fewer letter position errors when the word that resulted from the error required letter form change, than when the word resulting from transposition includes the same letter forms as the original word. Put differently, we ask whether migrations are less likely when they create an illegal orthographic sequence.

In the first stage of word reading, the stage of orthographic-visual analysis, two separate processes take place: abstract letter identification, and letter position encoding [6–8,20,25]. Abstract letter identity, ALI, is independent of information that is irrelevant to word reading, such as information about size, case, and font [1,4,5,9,23,26,34,11] (see Rapp et al. [27] for a review). If letter form is encoded (as part of the ALI or as an additional source of information) during the early stages of Arabic word reading, and is not discarded when the ALI is created, then letter form should affect reading, provide information on letter position, and thus modulate errors in LPD.

Table 1
Arabic letter forms

| Final non ligated | Final ligated | Medial ligated | Initial (or medial non ligated) | IPA |
|---|---|---|---|---|
| ا | ـا | ـا | ا | a |
| ب | ـب | ـبـ | بـ | b |
| ت/ ة | ـت/ ـة | ـتـ | تـ | t |
| ث | ـث | ـثـ | ثـ | θ |
| ج | ـج | ـجـ | جـ | ʤ/g |
| ح | ـح | ـحـ | حـ | ħ |
| خ | ـخ | ـخـ | خـ | x |
| د | ـد | ـد | د | d |
| ذ | ـذ | ـذ | ذ | ð |
| ر | ـر | ـر | ر | r |
| ز | ـز | ـز | ز | z |
| س | ـس | ـسـ | سـ | s |
| ش | ـش | ـشـ | شـ | ʃ |
| ص | ـص | ـصـ | صـ | sˤ |
| ض | ـض | ـضـ | ضـ | dˤ |
| ط | ـط | ـطـ | طـ | tˤ |
| ظ | ـظ | ـظـ | ظـ | zˤ |
| ع | ـع | ـعـ | عـ | ʕ |
| غ | ـغ | ـغـ | غـ | ʁ |
| ف | ـف | ـفـ | فـ | f |
| ق | ـق | ـقـ | قـ | q |
| ك | ـك | ـكـ | كـ | k |
| ل | ـل | ـلـ | لـ | l |
| م | ـم | ـمـ | مـ | m |
| ن | ـن | ـنـ | نـ | n |
| ه | ـه | ـهـ | هـ | h |
| و | ـو | ـو | و | w/u |
| ي | ـي | ـيـ | يـ | j/i |

A somewhat similar property of the Hebrew orthography was reported to affect reading in LPD (and in neglexia). Five of the letters in Hebrew have two different forms: one form is used only at the end of the word, and the other form is used in all other positions. Friedmann and Gvion [14] analyzed the rate of letter transposition that involved these final form letters when they appeared as final letters in the target word and moved to a non-final position, and when they appeared as penultimate and moved to final position, and compared these rates to such errors in letters that do not change form, in Hebrew-speaking individuals with acquired or developmental LPD. The results were clear cut: there were almost no errors that caused letter-form change, i.e., letters in final form did not change their position, and letters in non-final form did not move to final position if this required a letter form change. Similarly, Friedmann and Gvion found that neglect errors were also affected by letter form. Additions did not occur after a letter in final form (because this would require a final form to appear in middle position), and no omissions occurred after a letter in non-final form if it was a letter that had to appear in a different form as a final letter (because this would require a nonfinal form letter to appear in final position).

Arabic orthography is even more promising for a research of this type: whereas in Hebrew only 5 letters change form, and only letters in the final position (which very rarely migrates in LPD) change form, in Arabic all the letters change their form (or ligation), and different positions in the middle of the word also yield different forms, and some letters have as many as 4 different forms. Thus, Arabic orthography offers a unique window through which we can look at the creation of abstract letter identity and of the effect of orthographic constraints such as position-dependent letter forms on the manifestation of LPD, questions that are left unanswered if only English-like orthographies, which include no such letter form changes, are studied. (See [14] for a discussion of the possible effects of capital letters on reading in dyslexia).

The question of whether there are fewer letter position errors when the word that results from the error requires letter form change than when it includes the same letter forms as the original word was tested in 10 individuals with developmental LPD by comparing migration errors that do and do not change letter form. Another related question was whether, because of the relationship between letter position and letter form in Arabic, the general rate of letter position errors is lower than in languages in which middle letters do not change their form. This was tested in a bilingual Arabic-Hebrew reader with acquired LPD, whose letter position errors in Arabic and Hebrew were compared (and for whom we also tested the effect of letter form on letter position errors).

## 2. Developmental LPD

### 2.1. Method

#### 2.1.1. Participants

The participants with developmental LPD were ten Arabic-speaking individuals. They were speakers of Palestinian Arabic and readers of Standard Arabic, 3 girls and 7 boys, aged 10;0–17;5 ($M = 12;8$) at the time of the testing.[1] All of them lived in the Galilee, in the north of Israel. None of them had a history of brain lesion, neurological disease, or loss of consciousness. They all studied in regular classes in regular schools, and none of them had language disabilities (SLI). The teachers and parents of all these participants reported them as having learning problems at school, and this was their reason for referring them to us for diagnosis, though they had not been diagnosed with dyslexia before we tested them.

They were diagnosed with LPD on the basis of the Arabic TILTAN screening test [12] which includes 207 words, selected so that the word list can detect the various types of dyslexia – including words that, when read through the grapheme-to-phoneme conversion route, can be read as other words, for the detection of surface dyslexia, words that can be read as different words following the migration of a letter from a word above or below, for the detection of attentional dyslexia, words with many orthographic neighbors for detecting visual dyslexia, words with a lexical potential for omission or substitution on the left or on the right, for the detection of neglexia, abstract words and function words for the detection of deep dyslexia, and, crucially, 38 migratable words with a lexical potential for middle letter migration, for the detection of LPD.

For each of the ten participants, the main error type was migration of middle letters. On average, they made migration errors on 18.7% of the migratable words in the list (range 8%–37%, SD = 9.4%), all of them in middle positions. They had very few errors that were not letter-position errors, and were clear cases of selective LPD. However, because 6 of them also had some degree of vowel letter dyslexia, which also involves vowel migrations, but results from a deficit in the sublexical route [21], we included in the analysis only words in which the migration is of two consonants.

We also administered a lexical decision task in which 40 letter sequences were presented for lexical decision, of which half were real words, and half were migratable nonwords (like "talbe"), and a migratable word comprehension task, in which the participants saw 10 migratable words, each word presented with two pictures, one matching the word and the other matching the migration counterpart of the word. These tasks were administered to seven of the participants (the last seven in Table 2), and also showed a clear problem with letter position encoding: On the average, in the lexical decision task they accepted 51.4% of the migratable nonwords as words (range 20%-90%, SD = 27.5%). In the word-picture matching task they performed at chance, pointing only 61.4% of the time to the correct picture (range 20%-90%, SD = 22.7%). These tasks also support the diagnosis of LPD, because they indicate that the deficit is located in the reading input stage, and affects word identification and comprehension even when reading aloud is not required.

#### 2.1.2. Material

For the assessment of the effect of letter form on migration errors, each participant read a list of 122 migratable words in Standard Arabic,[2] 4–6 letters long.

Friedmann and Gvion [13,14] and Friedmann and Rahamim [15,16] reported three main characteristics

[1] We tested an additional adolescent with developmental LPD, who showed the same pattern of results. However, because she also had a hearing impairment, we did not include her in the study, as it is not clear how a hearing impairment affects the performance with respect to the research questions.

[2] The spoken language of our participants was Palestinian Arabic, a language that is not written (as is the case for all spoken varieties of Arabic). Standard Arabic is the written language, and the language used in formal contexts, which differs from Palestinian Arabic in lexical items (as well as in certain aspects of phonology and syntax). Thus, the participants grew up speaking Palestinian Arabic, but learned to read the related but not identical form, Standard Arabic. Because of this diglossic situation [10,29], we verified that the words we used in Standard Arabic were known to the participants.

that affect letter migration in acquired and developmental LPD in Hebrew. Firstly, significantly more letter position errors occur in *migratable words*, i.e., words in which a letter migration creates another existing word (like *form* and *from* in English). Secondly, most of the letter position errors occur in *middle positions*, whereas the first and final letters are relatively immune to migrations. Finally, more letter position errors occur when the target word is *less frequent* than its migration counterpart.

Thus, all the words in our list were created according to these three principles: they were migratable words, with a potential for middle letter migration, and the selected migratable word from each migratable word pair was always the less frequent word (according to the judgment of three native speakers of Arabic).

Each of these words belonged to one of four types with respect to the change in letter form following a letter position error: letter form changing, final-letter form changing, ligation changing, and same form.

The *form change* list included 24 words in which a middle letter migration causes a change in the form of a middle letter, such as جاهز - جهاز (GAHZ-GHAZ), namely, words in which a letter position change of the middle letters, keeping the original letter forms, would create an orthographically illegal sequence (in the common fonts). This was created by selecting migratable words in which one middle letter was a letter that had different middle letter forms when ligated and when not ligated to the preceding letter: these letters have the following form when ligated: ـعـ, ـغـ, ـهـ, whereas their respective non-ligated forms are: عـ, غـ, هـ. These letters appeared before or after one of the letters that do not ligate to the following letter (ا, د, ذ, ر, ز, و), so that when the middle letter order changed, it created a word in which the moved letter changed its ligation status and hence its form.

The *final letter form change* list included 16 words in which a middle letter migration creates a change only in the form of the final letter (and only change of ligation in middle letters). For example, a change in the order of the letters رق (RK) in the word برقة yields the word بقرة (BRKH-BKRH), in which the final letter H changes its form from ـة to ة because it now appears following the non ligating letter ر (R).

The *ligation change* list included 42 words in which a middle letter migration creates a change in the ligation status of a letter, without changing its form, like شارع – شراع (ŠARʕ – ŠRAʕ).

The *same form* list included 40 migratable words in which a middle letter migration does not cause any change in letter form, like يصنع – ينصع (INCNʕ–ICNCʕ). See the Appendix for examples of each of the four word types.

The stimuli of the four conditions were randomly ordered, and displayed on a white page, one below the other, in 14 pt. font with double vertical spacing between the words.

*2.1.3. Procedure*

Each participant was tested separately in a quiet room. The participants were asked to read each word aloud, and no time limit was set. The analysis was made on their first responses to each word.

The comparison between the performance in two experimental conditions for each participant was done using a chi-square test when the expected cell frequencies were equal to or greater than 5, and using Fisher's exact test when the expected frequency for one or more cells was smaller than 5. The comparison between conditions at the group level was conducted using a $t$-test. An alpha level of 0.05 was used.

*2.2. Results*

The results indicated that letter form had a crucial effect on the rate of letter position errors in LPD. All participants presented the same pattern: they made fewer letter position errors when the change of position caused change in letter form than when the position error did not change the letter form.

As seen in Table 2, whenever the letter position error created a form change, either of the transposing middle letters themselves or of the final letters, almost no position errors occurred. Only 5 errors occurred within a total of 240 words from the form-changing list. The participants not only refrained from moving letters that would change their own form, but also refrained from middle letter position errors when they created form change in the final letter, which did not move itself, but was affected by middle letter migration: they made only 6 such errors out of a total of 160 words (4%). This is in contrast with the very high letter position error rate, 40%, when the middle letters that transposed did not change their letter form or letter ligation. Some of the participants even had more than 80% errors on these words.

In the words in which migration changed only the ligation of middle letters, without changing the letter form, the participants made 10% errors. Namely, not only the change of letter form itself but also a change of its ligation reduced the letter position error rate.

Table 2
Number of migration errors in the 4 types of migratable words

| Number of words | Form change $n = 24$ | Final letter form change $n = 16$ | Ligation change $n = 42$ | Same form $n = 40$ |
|---|---|---|---|---|
| RO | 0 | 0 | 4 | 34 |
| SA | 1 | 2 | 7 | 33 |
| AB | 0 | 0 | 9 | 35 |
| HM | 1 | 0 | 2 | 5 |
| AS | 0 | 1 | 1 | 10 |
| MX | 1 | 0 | 3 | 9 |
| MS | 0 | 1 | 2 | 5 |
| SHS | 1 | 0 | 4 | 8 |
| HI | 1 | 2 | 10 | 14 |
| AL | 0 | 0 | 0 | 8 |
| *Total %errors* | *2%* | *4%* | *10%* | *40%* |

The letter position error rate was significantly higher in words in which letter position errors do not change letter form than in words in which the form of the transposing letters changes, $t(9) = 3.77, p = 0.002$, words in which the form of the final letter changes, $t(9) = 3.69$, $p = 0.003$, and words in which the ligation changes, $t(9) = 3.49$, $p = 0.003$. More errors were found in words in which migration only changes the ligation of letters than in words that change middle letter form, $t(9) = 3.26, p = 0.005$. Furthermore, on the individual level, each individual participant made more errors on the words with no form change than on the two word types that involved letter form change, this was significant using Fisher's exact test at the $p < 0.03$ level for nine participants, and $p = 0.10$ for the participant who had the fewest errors. Similarly to the reports from previous studies of LPD [13–15], the migrations occurred almost exclusively in middle letters, and only two errors occurred that could be interpreted as a transposition of first and second letters (but could also be interpreted as a migration of a diacritical marking).

The very high percentage of middle letter migration errors in migratable words that did not change letter form or letter ligation, 40% in average, is significantly larger than chance (using binomial distribution) for some of the participants, indicating that they did not randomly guess the order of the middle letters, rather they actually reversed them most of the time. This is probably because the letter position encoding function did not provide the lexicon with information about the position of the middle letters, and the lexicon retrieved the most accessible word that matched the partial information about the word. The fact that the participants were usually wrong was a result of our decision to always use the less frequent word of each migratable word pair as the target word.

## 3. Acquired LPD in a reader of Arabic and Hebrew

To compare the rate of letter position errors in a language in which letter forms vary according to letter position and in a language in which there are no such letter forms (only 5 final forms that are not affected by middle letter migrations), the best comparison would be a within-subject comparison. Thus, to compare the error rates between the languages and to further assess the effect of letter form on letter position errors in LPD in its acquired form, we tested IM, a bilingual reader of Arabic and Hebrew who had acquired LPD.

### *3.1. Participant*

IM is a 28 year-old man who sustained a traumatic brain injury with left parietal compressed skull fracture. He was a proficient speaker of both languages – he spoke Arabic at home, he served in the Israeli army where he spoke Hebrew, and spoke both languages at work and in social contexts. He had no premorbid history of reading, writing, or language disorders in either of the languages. Following the brain injury, he had dyslexia and dysgraphia in Arabic and in Hebrew.[3]

In reading, his main error type was migration of middle letters. His comprehension of single written migratable words in Hebrew, even when the test did not to require reading aloud, also showed middle letter migration errors, and hence, indicated a deficit at the input stage of reading. He made no phoneme order errors in spontaneous speech. In writing Hebrew and Arabic he also mainly made errors of middle letter order, some migrations of diacritics, and only a single position error of an exterior letter.

[3]We thank Galit Ben-Or for her help with the testing of IM.

### 3.2. Method

We asked IM to read a list of 81 Hebrew migratable words and a list of 111 Arabic migratable words on the same day.[4] The words were presented one above the other, with double spacing between lines, and IM was asked to read them aloud, with no time limits.

### 3.3. Results

In the Hebrew list of migratable words, IM made 23% middle letter position errors (19 errors out of 81 words),[5] whereas in Arabic he only made 9% (10 errors out of 111 words) middle letter position errors. Crucially, his letter position errors in Arabic occurred only in migratable words in which a migration did not change letter form (or ligation). When we counted only the same-form migratable words, he made 28% letter position errors of the target same-form words. He made no letter position errors in form-changing words. Thus, his results with respect to the way in which form-changing letters affect letter position errors were the same as those we found for the participants with developmental LPD: he made letter position errors only when these errors did not involve a letter-form change.

An important result emerges from the comparison of IM's error rates in Arabic and in Hebrew. When considering the full list of migratable words, he made significantly fewer letter position errors in Arabic than in Hebrew (9% compared to 23%), $\chi^2 = 7.62, p = 0.006$. Here, it is crucial to recall the difference between Arabic and Hebrew: whereas in Arabic only some of the migratable words preserve letter form following migration, in Hebrew all middle letter migrations preserve letter form. When calculating IM's percentage of letter position errors in Arabic only out of the non-form-changing migratable words, his error rate was 28%. Thus, when considering only the migratable words that do not change letter form, the migration rates in Arabic and Hebrew become very similar (28% and 23%, $\chi^2 = 0.25, p = 0.62$). Therefore, although IM had the same deficit in the two languages, with a tendency to make letter position errors in approximately a quarter of the words, letter-form in Arabic significantly reduced his general error rate, and blocked errors in words in which letter position errors cause letter form change.

[4]This was crucial because he was recovering quickly, and two weeks later, when we came to administer more tests, he was already reading almost without errors, so we report only the data collected on that day.

[5]His main error type was letter position errors. In addition, he made a few other errors: 1 omission and 1 substitution of a letter in Hebrew, and 4 omissions/shortening of vowels in Arabic.

## 4. Discussion

This study demonstrated, for the first time, letter position dyslexia in Arabic, in both developmental and acquired forms. It showed that letter form has a crucial effect on reading in LPD. Whereas letter migrations that did not cause letter form change occurred very frequently (40% on the average, and up to 88% errors for some of the participants with developmental LPD), almost no errors of letter position were made when they would have caused a change in letter form, namely, when transposing the letters using the original letter forms would create an orthographically illegal word.

This effect existed both when the middle letter transposition would have changed the form of the transposing letters, and when it would have changed the form of the final letter, which was not involved in the transposition. Even when the letter position errors would have caused only a change in the ligation of the letters, the rate of letter position errors was reduced, albeit to a smaller degree than for the letter-form change words.

Thus, letter form reduces migrations in LPD. Although prima facie it seems that an orthography with several letter forms for each letter would be harder to read and learn, the results from IM, a bilingual reader of Arabic and Hebrew with acquired LPD, indicate that the general rate of letter position errors is actually lower in Arabic than in Hebrew. This was indicated by his significantly higher error rate in Hebrew than in Arabic, alongside with the similarity in error rates when only non-form-changing migratable words were counted in both languages. Thus, the existence of varying letter forms actually reduces letter position errors. For example, when reading a book or an article (in which there are migratable words of all kinds, i.e., changing and non-changing letter forms), orthographies with position-related letter forms are expected to yield lower migration error rates than orthographies in which middle letters have the same form in all positions.

These results have straightforward implications for the diagnosis of LPD in Arabic, and probably for other languages with position-related letter forms. To detect LPD in these languages, the words that should be included in a diagnostic word list should be words with a lexical potential for middle letter migrations that do not change the letter form.

The findings of this study also have theoretical implications with respect to the early stages of word reading. The dissociation found between good letter identification, reflected in the absence of letter identity errors, and poor letter position encoding, reflected in letter position errors, supports the modularity of the functions of the orthographic-visual analysis system (see Fig. 1). It indicates that letter identification and letter position are two separate functions, which can be selectively impaired. This is so even in the developmental form of LPD. Although the participants had developmental dyslexia, they mastered the identification of letter forms and the orthographic rules that determine them, and were able to use this information for anchoring letters to their positions. These results are consistent with Friedmann and Gvion's [14] results, according to which not only (Hebrew-speaking) individuals with acquired LPD, but also those with developmental LPD showed a significant effect of final letter form on letter position error rate. This finding not only indicates that letter identification and letter position encoding are dissociable, but also that letter identification can develop normally even when letter position encoding is selectively impaired.

### 4.1. *Letter form information is encoded in the ALI*

The other theoretical implication relates to the encoding of letter forms. During the identification of abstract letter identity, some information about letters that is irrelevant to word reading such as size and font is discarded. The results indicate that letter form information is not discarded. It is encoded and used. How is it encoded? We suggest that letter form information is encoded as part of the abstract letter identity, ALI [1, 4,5,9,23,26,34,11] (see Rapp et al. [27] for a review). One possibility is that it is encoded as an additional type of information in the abstract letter identity, namely, all letter forms of the same letter are mapped onto the same ALI but with indication of the form they appeared in. Another possibility is that the different forms of the same letter have different abstract letter identity units. Even if separate ALIs are created for different letter forms, the different forms should still be identified in the orthographic input lexicon as instances of the same letter.[6] This is because Arabic is a Semitic language, organized by tri-consonantal roots. The same letters of the root always appear in the same relative order, but they can appear in various positions in words of the same root, depending on the inflection and template. (For example, the letter H of the root HRB can be incorporated as the initial letter in the word HARABA, ran-away, or as a middle ligated letter in the word YAHRUBU, run-away). In order to identify the morphological relation between these words, the identification of various forms of the same letter is required. If the lexicon stores morphemes rather than morphologically complex words [2,3,17,22,31–33], then it has to be able to store the same abstract root (or stem) for different morphologically complex words that include the same root, and therefore has to have the roots abstracted away of letter forms.

### 4.2. *How are letter-form changing migrations blocked?*

Another implication of the results relates to the way form-changing letter position errors are blocked. To describe how the difference in letter forms prevents letter position errors, we start by explicating our basic assumptions regarding LPD. As discussed in Friedmann and Rahamim [15], the clear effect of frequency on letter position errors indicates that it is not the case that the relative position of middle letters is encoded incorrectly (because then the lexicon would have activated an incorrect word, regardless of its frequency), but rather that the orthographic-visual analyzer produces an output that is underspecified with respect to the position of middle letters. When the orthographic input lexicon receives this partial information, it retrieves the first word that matches the partial information, and because it is ordered according to frequency, the lexicon is more likely to retrieve first the more frequent word that matches this information. This was the case with the participants in the current study as well. The effect of frequency on their reading could be seen in the finding that some of them had more letter position errors on non-form-changing migratable words than expected by chance. This rate resulted from the properties of the presented words: we presented only the migratable words that were the less frequent words of each migration pair. This finding, indicating that the reading of

[6] A mechanism such as the one put forward by Polk and Farah [26] cannot explain how various letter forms are identified as instances of the same letter. Polk and Farah [26] suggested that two letters are mapped onto the same ALI unit if they appear in the same context of other letters; for example, if a letter in the same word appears once in capital and once as a small letter ("Word" and "word"), the two occurrences are mapped to the same ALI (See [14] for a discussion of the difference between letter form and case). In Arabic, a letter always takes the same letter form in the context of the same letters; different letter forms never appear in the same context, because the same letters before and after the letter in the same relative order would always require the same letter form. Thus, such mechanism could not account for how various letter forms in Arabic are mapped to the same ALI.

our participants was affected by frequency, shows that they have the same underlying deficit as the previously reported LPD cases: their letter position information was missing, rather than incorrectly encoded.

Under the assumption of underspecified middle letter positions, there are two possible mechanisms by which letter form modulates letter position errors. One possibility is that the orthographic input lexicon encodes words with their letter forms. When a word like جهاز (GHAZ, gihaz, equipment) is read, it includes a middle-form H, so the partial information that reaches the lexicon, which includes the letter form, matches only this word in the lexicon, and not its migration counterpart جاهز (GAHZ, gahez, ready) which, although an existing word, includes an initial-form H (in contrast with the middle form of H in GHAZ which is used after a nonligating letter). This option explains why non-form-changing migrations can occur, but the letter strings that result from form-changing migrations do not exist in the orthographic input lexicon, and are thus never made. This possibility however, is not completely plausible if one assumes that the lexicon in Arabic includes morphemes rather than whole morphologically complex words because the same root morpheme (or stem) is mapped to different letter forms according to its position in the complex word. Therefore, it seems more reasonable that the lexicon would store the morphemes abstracted away from their letter forms, because the letter form of the same root letter can change in different inflections and templates. Moreover, if it were only the lexicon that prevented letter position errors that create orthographically illegal letter sequences, letter position errors violating letter form would have been blocked in words, but would still occur in nonwords.[7]

Another possibility, which fits the morphological considerations better, is that the first stage of orthographic-visual analysis encodes letter identity, still including the letter form, and letter position. This information then enters a pre-lexical mechanism, possibly a graphemic input buffer, which holds the information for a short time, and checks it for orthographic violations.[8] This might also be the stage at which the initial morphological decomposition takes place [28, 30]. If an orthographic violation such as initial letter form in middle ligating position is detected at this stage, this representation crashes, and the information cannot be transferred to the next stages, the lexicon and the grapheme-to-phoneme converter. Within a situation of underspecification of middle letter position, one may assume that all possible options of middle letter position arrive in this checking buffer, but the illegal ones crash. This would yield the observed pattern of there being no errors that violate the orthographic well-formedness rules. This graphemic input buffer is sensitive to letter forms, but the next stage, the lexicon, no longer needs to have access to letter forms, so letter form information may be discarded after the stage of orthographic checking.

Thus, this study indicates that the existence of letter forms that are determined by the relative position of the letter within the word modulates letter position errors in Arabic LPD, by blocking letter position errors that change letter form. This, in turn, reduces the general error rate in LPD in Arabic, which has position-dependent letter forms for middle letters, in comparison to Hebrew, a language with a similar orthography and morphology, which does not have position-dependent forms for middle letters. These findings support the existence of two separate functions of the orthographic-visual analysis system, abstract letter identification and letter position encoding, which develop separately and can be selectively impaired. The effect of letter form on letter position errors in LPD in a morphologically rich orthography suggests that a pre-lexical mechanism exists, which receives the information from the first stage of orthographic-visual analysis, which still includes letter form information, checks the well-formedness of the input string.

**Acknowledgments**

This research was supported by the Israel Science Foundation (grant no. 1296/06, Friedmann), and by the

[7] We only have data from the oral reading of 16 migratable nonwords per participant for 3 participants and for 12 migratable nonwords for 7 participants, but within this small number of nonwords, they had an average of 35.8% migration errors (SD = 21.9%). All their errors were migrations of letters in middle positions, and they made no violation of letter form. Clearly, a broader investigation of the reading of migratable nonword reading with and without form change in LPD is desirable.

[8] This mechanism may also conduct this checking on graphemic units that are smaller than the whole word. Howard [19] and Hall, Humphreys, and Cooper [18] suggested the encoding of multiletter units specifying the visual relations between frequent, common adjacent letters or letter groups, particularly those that might act as graphemic units. The checking of two adjacent letters would suffice to detect letter form violations.

## Appendix

Examples for words in each of the four conditions – the target word and the lexical result of middle letter migration.

| Word type | Target word | Expected result of middle letter migration |
|---|---|---|
| Form change | ترغب | تغرب |
| | جاهز | جهاز |
| | شعار | شاعر |
| | مزعوم | معزوم |
| Final letter form change | قرفة | قفرة |
| | واسع | وساع |
| | شفرة | شرفة |
| | برقة | بقرة |
| Ligation change | مرصوف | مصروف |
| | شراع | شارع |
| | يرقب | يقرب |
| | تجوب | توجب |
| Same form | مجتر | متجر |
| | بضعه | بعضه |
| | ينصع | يصنع |
| | شفقة | شقفة |

ARC Centre of Excellence in Cognition and its Disorders (CCD), Macquarie University. We thank Anne Castles, Dror Dotan, Aviah Gvion, Sachiko Kinoshita, Lyndsey Nickels, Julia Reznick, and Maya Yachini for their helpful comments.
